# Tangential Field Radiotherapy for Breast Cancer—The Dose to the Heart and Heart Subvolumes: What Structures Must Be Contoured in Future Clinical Trials?

**DOI:** 10.3389/fonc.2017.00130

**Published:** 2017-06-19

**Authors:** Marciana Nona Duma, Anne-Claire Herr, Kai Joachim Borm, Klaus Rüdiger Trott, Michael Molls, Markus Oechsner, Stephanie Elisabeth Combs

**Affiliations:** ^1^Department of Radiation Oncology, Technical University of Munich (TUM), Munich, Germany; ^2^Center for Stereotactic and Highprecision Radiation Therapy (StereotakTUM), Technische Universität München (TUM), Munich, Germany; ^3^Department of Radiation Sciences (DRS), Institute of Innovative Radiotherapy (iRT), Helmholtz Zentrum München, Munich, Germany; ^4^Medical School, Technische Universität München, Munich, Germany; ^5^Cancer Institute, University College of London, London, United Kingdom; ^6^Technische Universität München, Munich, Germany

**Keywords:** breast cancer, heart, tangential field, left anterior descending artery, radiotherapy

## Abstract

**Background and purpose:**

The aim of the present study was to evaluate if it is feasible for experienced radiation oncologists to visually sort out patients with a large dose to the heart. This would facilitate large retrospective data evaluations. And in case of an insufficient visual assessment, to define which structures should be contoured and which structures can be skipped as their dose can be derived from other easily contoured structures for future clinical trials.

**Material and methods:**

Planning CTs of left-sided breast cancer patients treated with 3D-conformal radiotherapy by tangential fields were visually divided into two groups: with an estimated high dose (HiD) and with an estimated low dose (LoD) to the heart. For 46 patients (22 HiD and 24 LoD), the heart, the left ventricle, the left anterior descending artery (LAD), the right coronary artery, and the ramus circumflexus were contoured. A helper structure (HS) around the LAD was generated in order to consider if contouring uncertainties of the LAD could be acceptable. We analyzed the mean dose (Dmean), the maximum dose, the V10, V20, V30, V40, and the length of the LAD that received 20 and 40 Gy.

**Results:**

The two groups had a significant different Dmean of the heart (*p* < 0.001). The average Dmean to the heart was 4.0 ± 1.3 Gy (HiD) and 2.3 ± 0.8 Gy (LoD). The average Dmean to the LAD was 26.2 ± 7.4 Gy (HiD) and 13.0 ± 7.5Gy (LoD) with a very strong positive correlation between Dmean LAD and Dmean HS in both groups. The Dmean heart is not a good surrogate parameter for the dose to the LAD since it might underestimate clinically significant doses in 1/3 of the patients in LoD group.

**Conclusion:**

A visual assessment of the dose to the heart could be reliable if performed by experienced radiation oncologists. However, the Dmean heart is not always a good surrogate parameter for the dose to the LAD or for the Dmean to the left ventricle. Thus, if specific late toxicities are evaluated, we strongly recommend contouring of the specific heart substructures as a heart Dmean is not highly specific.

## Introduction

The heart is probably the most radiosensitive organ in the human body. Long-term follow-up of the Japanese A-bomb survivors demonstrated that a mean body dose (and thus mean heart dose) of 1 Gy increased the mortality from heart diseases by 14% (1). Follow-up studies in patients treated for various malignant and non-malignant diseases yielded similar risk values (2). Careful analysis of the pathologies of radiation-induced heart diseases after mantle field radiotherapy of patients for Hodgkin’s disease (thus receiving a near-homogeneous dose to their hearts) demonstrated that five different radiation-induced heart diseases were diagnosed, namely, pericarditis, myocardial fibrosis, coronary atherosclerosis leading to myocardial infarction, conduction defects such as bundle branch blocks and valvular insufficiency. Each of these manifestations of cardiac radiation injury occurs in different substructures of the heart, follows different pathogenic pathways, and may have different dose dependence. This means, however, that different manifestations of cardiac radiation damage would be expected to occur after different anatomical dose distributions, such as from adjuvant radiotherapy of breast cancer patients and that mean heart doses may not be a relevant dose criterion for estimating cardiac complications from particular treatment plans.

Notwithstanding this argument, large retrospective data have demonstrated a relationship between the delivered heart dose and major coronary events. A recent study by Darby et al. (3) analyzed the risk of ischemic heart disease in women after radiotherapy for breast cancer. They have found that the average mean dose (Dmean) to the heart of patients treated between 1958 and 2001 was 4.9 Gy with a significant correlation between the mean heart dose and major coronary events. However, no individual dosimetric data were available for this retrospective study. In order to assess the mean heart doses and the Dmean to the anterior descending coronary artery, the 2D-plans were recalculated on a “typical” patient in the Darby et al. study. Studies have also shown a direct link between radiation dose in the coronary arteries and the location of coronary stenosis (4).

Although heart dose from breast cancer radiotherapy has been significantly reduced over the past decades, parts of the heart may still be located in the radiation field in modern 3D-conformal radiotherapy (3D-CRT) (5–7). Hence, it is essential to select all patients, which, with conventional techniques, could receive a significant dose to critical structures of the heart and offer them a cardiac sparing radiotherapy.

However, contouring of all the heart subvolumes is time consuming. Moreover, it has to be considered that there may be clinically and dosimetrically significant interobserver variations in heart and heart subvolume delineations (8). Lorenzen et al. found substantial interobserver variation in the estimated dose of the left anterior descending artery (LAD), which even guidelines could not reduce. The coefficients of variation in the estimated doses to the LADCA were for Dmean 27% without and 29% with guidelines. For the heart, variation was little, especially when guidelines were used (9). Thus, it is essential to understand the dosimetric impact of contouring uncertainties in the LAD.

The aim of the present study was to evaluate if it is feasible for experienced radiation oncologists to visually sort out patients with a large dose to the heart. This would facilitate large retrospective data evaluations. And in case of an insufficient visual assessment, to define which structures should be contoured and which structures can be skipped as their dose can be derived from other easily contoured structures. More specifically, two questions were addressed: (1) is the visual evaluation a reliable indicator of mean heart dose and (2) is the mean heart dose a reliable indicator of the radiation exposure of the left anterior descending coronary artery/left ventricle?

## Materials and Methods

201 consecutive patients with left-sided breast cancer treated in our institution between March 2009 and November 2010 were identified.

These patients were all treated with 3D-CRT by tangential fields, half beam technique. Patients were placed on a breast board with the left arm above the head. The treatment planning was performed with the Eclipse Treatment Planning System (Varian Medical Systems, Palo Alto, CA, USA). All patients underwent a planning kVCT scan (Siemens Inc., Erlangen, Germany) with an axial slice thickness of 5 mm before treatment. The CT scans were not contrast enhanced. The treatment plans consisted of two opposing tangential wedged beams. Additional segments (1–2) were used to improve target dose homogeneity, if necessary. Both medial and lateral beams were wedged. The PTV prescribed dose was 50 Gy for the whole breast (ICRU reference point), followed by an electron boost of 10–16 Gy. All treatments were performed with daily single doses of 2 Gy.

**Figure 1:**
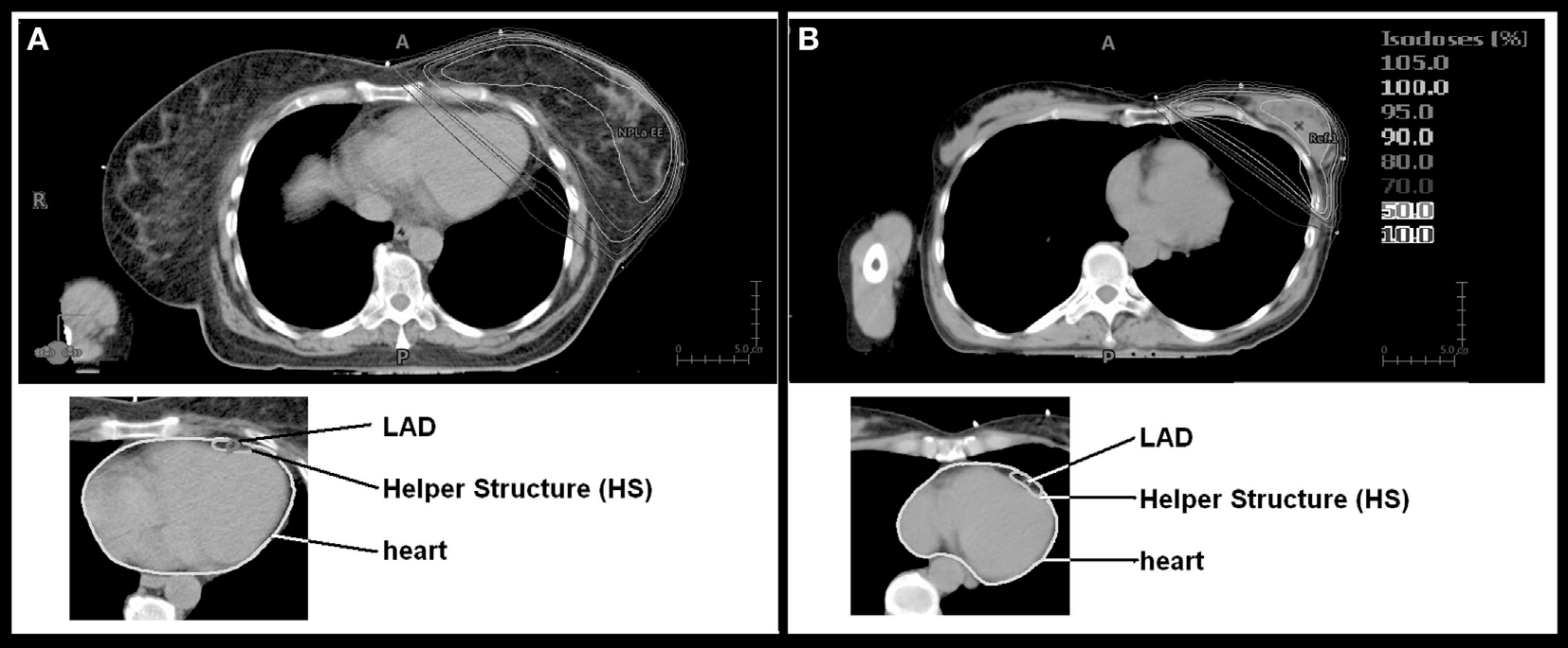
Isodose distribution and contouring of organs at risk. Depicted are the two patients with the largest [**(A)** mean dose (Dmean) 8.9 Gy] and the lowest [**(B)** Dmean 0.8 Gy] Dmean heart doses.

The planning CTs of the 201 patients were visually reviewed. The CTs with the calculated dose distributions for the whole breast radiotherapy (50 Gy) were presented to a radiation oncologist who was asked to assess whether the heart Dmean would be high or low. Figure 1 exemplarily depicts two CTs with the isodoses (10–105%) used in this study. No structures were superimposed on the CT scan. Assessment was performed visually. Taking for example the patient in Figure 1A, as a large part of the heart is within the 10% isodose, the patient was estimated by the radiation oncologist to have a high dose (HiD) to the heart. Thus, two groups were generated: one with a visually estimated HiD to the heart (86 patients) and the other one with an estimated low dose (LoD) to the heart (115 patients).

The treatment records of the 201 patients were reviewed and we excluded from this analysis: patients who underwent systemic therapy beforehand, patients who were treated for breast cancer relapse, patients who received irradiation to the locoregional lymph nodes, patients with mastectomy, and patients with concomitant bilateral breast cancer radiotherapy. This was done because in an ongoing study, we perform functional imaging to assess correlations between heart toxicities and dose distributions. From the remaining patients, the first 46 consecutive patients were chosen (24 from the LoD group and 22 patients from the HiD group) for this dosimetric study. The left ventricle, the LAD (LAD), the ramus circumflexus (RCX), and the right coronary artery (RCA) were retrospectively contoured according to the Feng et al. heart atlas (10). In order to test whether contouring uncertainties could be acceptable for the LAD, a helper structure (HS) with a width of 0.5 cm anterior–posterior and 1 cm left right around the LAD was generated (Figure 1). This was performed in order to test whether a significantly larger contouring of a very small region of interest can be safely performed.

For all contoured structures, dose volume histograms were analyzed. We assessed the minimum dose, maximum dose (Dmax), Dmean, absolute volume in cubic centimeters, V10 (the relative volume that receives 10 Gy or more), V20, V30, and V40. Additionally, the absolute volume V10, V20, V30, and V40 of the heart in cubic centimeters was assessed.

Since neither clinical nor radiobiological data provide a reliable data on the dose/volume dependence of radiation-induced atherosclerosis, different criteria of dose specification in the LAD were determined which would, in a second step, permit the determination of the anatomical relationship between local dose and local tissue injury. Therefore, in addition to V10 etc., also the absolute LAD length and the length of the LAD that lies within the 20 Gy isodose and within the 40 Gy isodose were evaluated. The statistical analyses were performed using SPSS Software for Windows version 20.0 (SPSS Inc., Chicago, IL, USA). All statistical tests were performed two-sided and a *p*-value <0.05 was considered to indicate statistical significance. Mean values are reported with SD, median values with range. Pearson correlations are presented.

## Results

In the 46 patients, the volume of the heart ranged between 471 and 1,013 cm^3^, the volume of the left ventricle between 141.2 and 275.3 cm^3^, the volume of the LAD between 1.1 and 2.6 cm^3^, the HS volume between 11.2 and 20.8 cm^3^, the RCX volume between 0.3 and 1.0 cm^3^, and the RCA volume between 0.7 and 1.8 cm^3^.

The median (range) Dmean/Dmax to the whole heart was 3.6 Gy (2.6–8.9 Gy)/49.3 Gy (47.7–51.6 Gy) for the HiD group and 2.6 Gy (0.8–3.5 Gy)/44.6 Gy (6.1–57.3 Gy) for the LoD group, respectively. The median (range) Dmean/Dmax to the left ventricle was 6.3 Gy (3.8–15.5 Gy)/49.2 Gy (46.1–51.4 Gy) for the HiD group and 4.0 Gy (1.0–6.0 Gy)/48.1 Gy (4.8–57.2 Gy) for the LoD group, respectively. Doses for the RCA or RCX were <1.0 Gy.

The two groups had a significant different Dmean of the heart (*p* < 0.001). Thus overall, the clinical assessment whether the heart will receive a HiD or LoD was good (Figure 1), yet, there was considerable overlap considering individual patients. In the HiD group 3 patients out 22 were wrongly estimated—Dmean within 0.5 Gy of the average Dmean heart of the LoD group. In the LoD group, 3 out of 24 patients were within 0.5 Gy of the average Dmean heart of the HiD group.

The overall average length of the LAD was 8.4 ± 0.8 cm (Mean ± SD). The length of the LAD that received 20 Gy/40 Gy was 4.5 ± 1.8 cm/2.9 ± 2.3 cm for the HiD group and 1.9 ± 1.7 cm/0.7 ± 1.1 cm for the LoD group, respectively.

**Figure 2:**
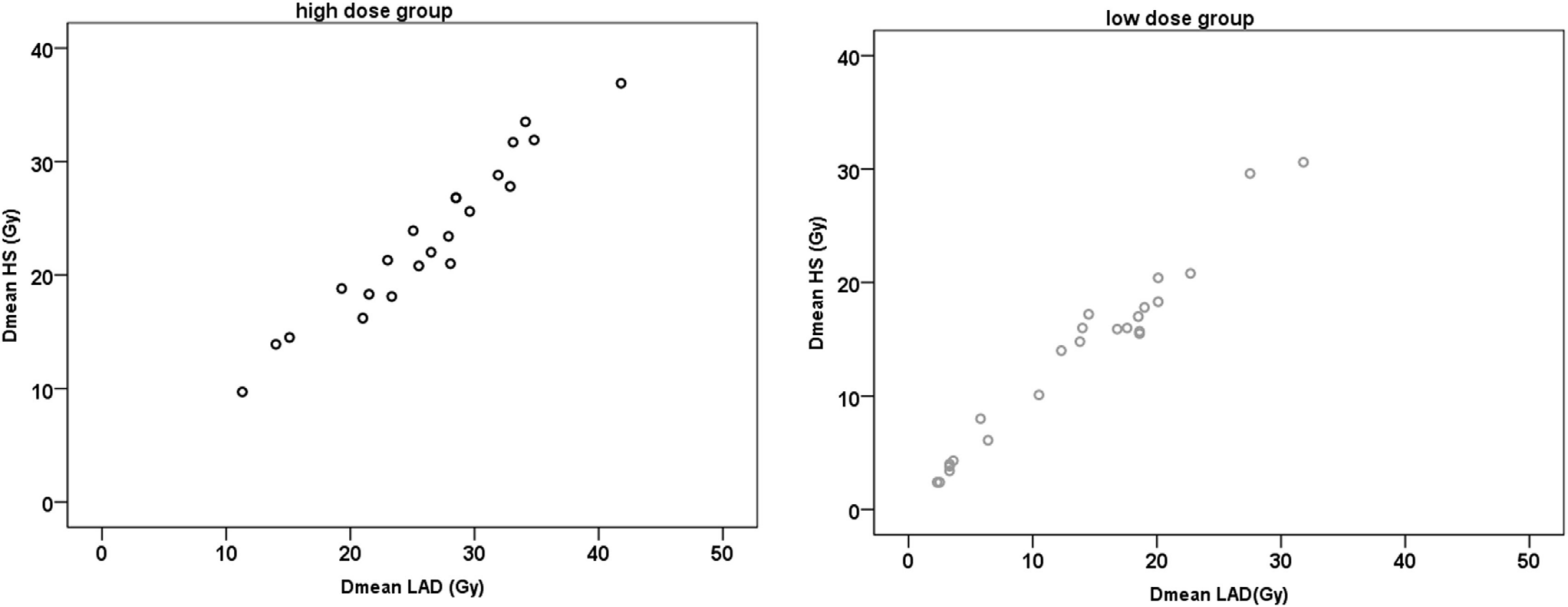
Scatter plots of mean dose (Dmean) left anterior descending artery (LAD) and Dmean helper structure (HS).

The average Dmean to the LAD/HS was 26.2 ± 7.4 Gy/23.3 ± 6.9 Gy for the HiD group and 13.0 ± 7.5 Gy/13.0 ± 7.2 Gy for the LoD group. In both groups, there were very strong positive correlations between the Dmean LAD and the Dmean HS (*r* ≥ 0.964; *p* < 0.001) (Figure 2).

**Figure 3:**
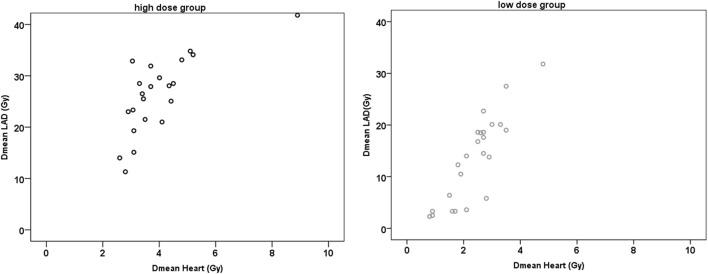
Scatter plots of mean dose (Dmean) heart and Dmean left anterior descending artery (LAD).

**Figure 4:**
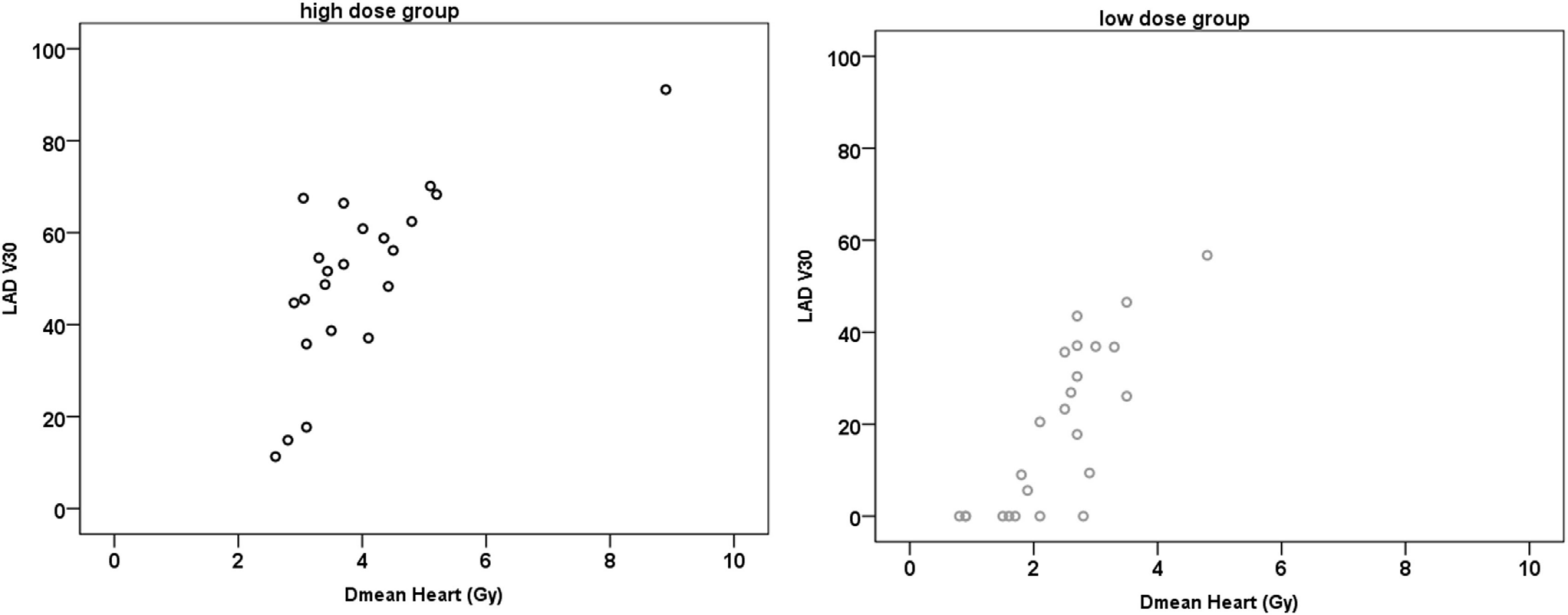
Exemplary scatter plots of the V30 (%) and the mean dose (Dmean) heart. In the low-dose (LoD) group, there are still 7/24 (29%) patients who have a left anterior descending artery (LAD) V30 of more than 30%. In the high-dose (HiD) group, this applies to 19/22 (86%) patients. Similarly, for the LAD V40, there are 6/24 patients in the LoD group who receive a dose of more than 40 Gy to more than 20% of the LAD and 19/22 in the HiD group (not depicted).

For both groups, there was a very strong positive correlation between the Dmean ventricle and the Dmean heart (*r* ≥ 0.902; *p* < 0.001) and a strong and very strong positive correlation between the Dmean heart and Dmean LAD/HS, respectively (HiD: *r* = 0.731/*r* = 0.724, *p* < 0.001; LoD: *r* = 0.834/*r* = 0.849, *p* < 0.001) (Figure 3). The correlation between Dmean ventricle and Dmean LAD was strong but not as strong as the one between Dmean heart and Dmean LAD (HiD: *r* = 0.642; LoD: *r* = 0.605; *p* ≤ 0.001). We found strong and very strong positive correlations between heart (relative volume) V10, V20, V30, and V40 and LAD V10, V20, V30, and V40 in the high as well as in the LoD group. Table 1 presents the absolute and relative V10, V20, V30, and V40 of the heart (Table 1). Figure 4 depicts exemplary scatterplots of the V30 of the LAD and the Dmean heart, which highlights the clinical problem. Despite significant correlation, the predictive value of the mean heart dose and high doses to critical volumes of the LAD may not be good enough.

**Table 1:** The V10, V20, V30, and V40 of the heart.

	Heart V10 (%)	Heart V10 (cm^3^)	Heart V20 (%)	Heart V20 (cm^3^)	Heart V30 (%)	Heart V30 (cm^3^)	Heart V40 (%)	Heart V40 (cm^3^)
**High-dose group**								
Median	6.1	46.3	4.9	36.7	4.1	30.1	3.0	22.8
Minimum	3.2	19.2	2.2	13.3	1.7	10.1	1.2	7.3
Maximum	19.0	129.6	16.8	114.5	15.0	102.7	11.9	81.1
**Low-dose group**								
Median	3.4	22.8	2.5	16.2	1.9	11.7	1.2	7.9
Minimum	0.0	0.0	0.0	0.0	0.0	0.0	0.0	0.0
Maximum	5.4	40.3	4.1	30.3	3.2	23.6	2.3	14.8

## Discussion

As breast cancer is the most common cancer in women and long-term survivorship is nowadays the rule, morbidity and mortality from radiation-induced heart disease has become a major concern in treatment planning. There is evidence that the risk of different potential late cardiac radiation injury depends on local radiation dose, which opens the possibility to reduce the risk by optimizing the dose distribution in the heart of the individual patient. This does, however, require detailed cardiac dosimetry for the individual patient as a basis for treatment plan optimization. Studies are available on modern heart dosimetry in breast cancer patients, revealing that even with contemporary treatment and planning techniques, some patients still receive important doses to the heart or to its substructures such as the LAD.

The aim of this study was to assess which structures should be contoured and which structures could be skipped as the dose could be derived from correlations with other structures.

The result of our study is that a visual assessment by experienced radiation oncologists often gives a reliable estimate of Dmean doses to the heart. There is a significant (*p* < 0.001) statistical difference in the Dmean to the heart between the two chosen groups. If the heart is not contoured due to workload, a retrospective visual examination of planning CTs from the department database with predefined range of isodoses (e.g., 10–105% isodose) would be very informative and the Dmean to the heart could be estimated for future patients.

However, some patients in our study were not perfectly matched to their group. We reviewed each patient’s CTs individually and found two main confounding factors.

First, our contouring—according to the Feng et al. (10) atlas—included the pericardium. Our group of patients, however, had some variations of the amount of epicardial fatty tissue. A simple scrolling through CT slices without definition of heart boundaries might visually group patients with larger epicardial fatty tissue into the LoD group as the pericardium is not always easily seen on every single CT slice. A large epicardial fatty tissue translates into a higher Dmean (since structures of the heart that were not visually considered, would now lie within the HiD region). These patients do not receive a large dose to the myocardium (left ventricle), but a significant dose to the LAD. Thus, either contouring of the heart including pericardium or contouring of all heart structures is necessary in these patients in order to estimate specific late toxicities.

Second, patients with a large dose to the heart on a few CT slices were wrongly categorized into to the HiD group. The HiD levels (40–50 Gy isodoses) extended into the heart on only very few CT slices (2–3 slices). Visually this can be misleading.

Focusing on mean heart dose solely, our findings suggest that visual grouping into the low heart dose category might be an acceptable way to eliminate detailed contouring in about half the patients with an error margin of 10%. In the other half of patients, detailed contouring should be recommended.

Yet, which structures should be contoured? Lorenzen et al. (9) found substantial interobserver variation in the estimated dose of the LAD, which even guidelines could not reduce. The spatial distance variation between the delineations was up to 7–8 mm. Thus, a structure like a HS might depict the whole uncertainties region. Overall, in our study, we found a very strong positive correlation between Dmean LAD and the Dmean HS (*r* ≥ 0.964; *p* < 0.001). As the HS represents the relative region in which we can assume that the LAD lays, we can conclude that contouring uncertainties might be acceptable. Even a rough contouring will be helpful for the clinician in order to assess the magnitude of dose to the LAD (i.e., 13 vs. 23 Gy Dmean in the two groups, respectively).

However, even significant correlations between values of dose specification are of limited use in the practice of treatment planning for the individual patient. Although the dose to the LAD correlates very strongly with the dose to the heart, even in the LoD group there are still patients who will receive a significant dose to the LAD (>30 Gy). To stress this point, in Figure 4, patients with the same Dmean heart (e.g., ≈2.7 Gy) had a LAD V30 value that ranged between 0% and approx. 50% in the LoD group and in the HiD group the LAD V30 even ranged from 10 to 70%. Thus, despite the strong positive correlation of the Dmean of the heart to the Dmean to the LAD, even in the LoD group, one-third of the patients will receive over 30 Gy to one-third of their LAD. In order not to skip any patients with a HiD to the LAD. we therefore recommend a contouring of the LAD/HS in all patients with left-sided breast cancer, independent of the estimated or calculated mean heart dose.

## Conclusion

A visual assessment could be reliable if experienced radiation oncologists have to assess whether a patient receives a higher or a lower Dmean to the heart. Even a rough contouring of the region LAD (i.e., the HS) provides clinically valuable information on the magnitude of the LAD dose. The Dmean heart is not always a good surrogate parameter for the dose to the LAD as it might underestimate clinically significant doses in one-third of the patients with a LoD. The Dmean heart is a good surrogate for the Dmean to the left ventricle, except for patients with a large epicardial fatty tissue. Thus, if specific late toxicities are evaluated, we strongly recommend contouring of the specific heart substructures as a heart Dmean is not highly specific.

## Consent Procedures

All patients gave their informed consent both informed and written before starting the radiotherapy that they will undergo CT radiotherapy treatment planning. Data from the CT radiotherapy treatment planning were retrospectively analyzed.

## Ethics Statement

This study was approved by the ethics committee of Klinikum rechts der Isar, Technical University Munich.

## Author Contributions

MD, A-CH, KB, KT, MM, and MO participated in the study design, contributed to the data collection, and drafted the manuscript. SC made important contributions in revising the content. All authors read and approved the final manuscript.

## Conflict of Interest Statement

The authors declare that the research was conducted in the absence of any commercial or financial relationships that could be construed as a potential conflict of interest.
